# 868. In Vitro Activity of Isavuconazole and Other Mould-Active Triazoles Against Aspergillus fumigatus With and Without cyp51 Alterations

**DOI:** 10.1093/ofid/ofac492.061

**Published:** 2022-12-15

**Authors:** Michael A Pfaller, Cecilia G Carvalhaes, Lalitagauri M Deshpande, Paul Rhomberg, Paul Rhomberg, Mariana Castanheira

**Affiliations:** JMI Laboratories, North Liberty, Iowa; JMI Laboratories, North Liberty, Iowa; JMI Laboratories, North Liberty, Iowa; JMI Laboratories, North Liberty, Iowa; JMI Laboratories, North Liberty, Iowa; JMI Laboratories, North Liberty, Iowa

## Abstract

**Background:**

Azole resistance in *Aspergillus fumigatus* (AFM) is mainly associated with mutations in *cyp51A* and its promoter region or its homologue *cyp51B*. We evaluated the *in vitro* activity of isavuconazole (ISC), itraconazole (ITC), posaconazole (PSC), and voriconazole (VRC) against 660 AFM collected during 2017–2020.

**Methods:**

Isolates from Europe (EU), North America (NA), Latin America (LA), and Asia-Pacific (APAC) were identified by MALDI-TOF and/or sequencing and tested by CLSI broth microdilution. CLSI epidemiological cut-off values (ECV) were applied. A PSC ECV of 0.5 mg/L was used. Non-wildtype (NWT) isolates to azoles were screened for alterations in the *cyp51* genes using whole genome sequencing.

**Results:**

Azoles had similar activities against 660 AFM isolates. Overall, AFM displayed WT MIC values to ISC (92.7%), ITC (92.9%), PSC (97.3%), and VRC (96.7%). Only 66 isolates (10.0%) were NWT to 1 or more of the azoles, and 32 harbored one or more alterations in the *cyp51* genes. Of these AFM, 29/32 (90.1%) were NWT to ITC, 25/32 (78.1%) NWT to ISC, 17/32 (53.1%) NWT to VRC, and 11/32 (34.4%) NWT to PSC. The most frequent alteration was CYP51A TR34/L98H, carried by 14 EU isolates, all NWT to ISC and ITC. Four isolates from NA carried the alteration I242V in CYP51A (all NWT to ITC; all WT to ISC). One NA isolate carried the CYP51A alteration G448S (NWT to ISC, VRC, and ITC) and one carried A9T (NWT to ISC). A single isolate from APAC carried a CYP51A G138C alteration and was NWT to all 4 triazoles. Multiple alterations in CYP51A were detected in 5 isolates: 4/5 NWT to ISC or ITC, 1/5 NWT to VRC, all WT to PSC. Alterations in CYP51B were noted in 7 isolates; 6/7 carried Q42L, 3 from NA (all NWT to ITC and 2 NWT to ISC), 2 from APAC (all NWT to VRC or ISC), and 1 from EU (NWT to ISC, ITC, and PSC). Among 34 NWT isolates without *cyp51* alterations, 32.4% were WT to ISC, 47.1% WT to ITC, 85.3% WT to VRC, and 82.4% WT to PSC.

**Conclusion:**

The majority of AFM were WT to azoles. Ten different *cyp51* alterations were detected in 32/66 NWT isolates. Only EU isolates harboured the environmental alteration TR34/L98H that was associated with a NWT phenotype to ISC and ITC. Alterations in AFM *cyp51* can have variable effects on the *in vitro* activity of the azoles that are best delineated by testing all triazoles.

**Disclosures:**

**Michael A. Pfaller, MD**, Pfizer: Grant/Research Support **Cecilia G. Carvalhaes, MD, PhD**, AbbVie: Grant/Research Support|Cidara: Grant/Research Support|Melinta: Grant/Research Support|Pfizer: Grant/Research Support **Lalitagauri M. Deshpande, PhD**, Melinta: Grant/Research Support|Pfizer: Grant/Research Support **Paul Rhomberg, BS, MT(ASCP)**, Cidara: Grant/Research Support|Pfizer: Grant/Research Support **Paul Rhomberg, BS, MT(ASCP)**, Cidara: Grant/Research Support|Pfizer: Grant/Research Support.

